# Hepatic Interferon-λ3 (*IFNL3*) Gene Expression Reveals Not to Be Attenuated in Non-Favorable *IFNL3* rs4803217 or *IFNL4* rs368234815 Minor Allele Carriers in Chronic Hepatitis C

**DOI:** 10.1371/journal.pone.0143783

**Published:** 2015-11-25

**Authors:** Ahmad Amanzada, Lars Reinhardt, Dorothea Fey, Elisabeth M. Zeisberg, Sabine Mihm

**Affiliations:** 1 Department of Gastroenterology and Gastrointestinal Oncology, University Medical Center, Georg-August University Goettingen, Goettingen, Germany; 2 Department of Cardiology and Pneumology, University Medical Center, Georg-August University Goettingen, Goettingen, Germany; 3 DZHK (German Centre for Cardiovascular Research), partner site Goettingen, Germany; University of Sydney, AUSTRALIA

## Abstract

Genetic polymorphisms in the region of the interferon-λ genes (*IFNL*) associate with clearance of hepatitis C virus (HCV) infection. One of these polymorphisms, *IFNL4* rs368234815, determines loss or gain of function of the *IFNL4* gene by frameshift variation. The very same and a second one, *IFNL3* rs4803217, are supposed to impact the expression of *IFNL3*: while *IFNL4* rs368234815 is suggested to modulate *IFNL3* transcription, *IFNL3* rs4803217 is thought to alter *IFNL3* mRNA stability. The latter process is believed to be partially driven by an HCV-induced ectopic expression of myosin heavy chain genes 7*B* and *7* and their co-expressed microRNAs mir499 and mir208B. These ideas are evidenced by functional investigations on peripheral blood mononuclear and hepatoma cells in culture. Our study aimed at exploring *IFNL3* gene expression in clinical samples, i.e., in *ex vivo* derived liver tissue from patients with chronic hepatitis C (n = 57) and various other diseases (n = 56). By applying an assay designed to specifically quantify *IFNL3* and discriminating paralogous *IFNL2* transcripts, *IFNL3* mRNA expression was not found to differ significantly between chronic hepatitis C and control samples. Among patients with chronic HCV infection, moreover, *IFNL3* rs4803217 or *IFNL4* rs368234815 minor alleles did not associate with reduced *IFNL3* gene expression. Finally, myosin heavy chain genes 7*B* and *7* and corresponding microRNAs mir499 and mir208B were not found activated in liver in chronic HCV infection. Of note, detectability of *MYH7* mRNA related to the procedure of liver biopsy sampling, as tissue obtained by direct punctation of the liver during laparoscopic inspection was less likely to contain *MYH7* transcripts than samples acquired by percutaneous punctation. In conclusion, data on *ex vivo* derived liver tissue samples argue against an attenuating impact of *IFNL3* rs4803217 or *IFNL4* rs368234815 minor alleles on hepatic *IFNL3* gene expression *in vivo*.

## Introduction

Infection with hepatitis C virus (HCV) affects an estimated 160 million people worldwide [1]. It causes chronic and progressive liver disease. In 2009, genome-wide association studies on main ethnic populations most convincingly have identified genetic variations in the region of the type III interferon-λ (IFN-λ/*IFNL*) genes to be correlated with clearance of infection [2–5]. Type III IFNs comprise *IFNL1*, *IFNL2* and *IFNL3* (also known as *IL29*, *IL28A* and *IL28B*, respectively) [6,7], and the recently discovered *IFNL4* [8]. As type I IFNs, type III IFNs confer antiviral activity. They utilize a unique heterodimeric receptor distinct from the type I IFN receptor, but they share signaling pathways with type I IFNs [9]. One prominent polymorphism shown to be closest correlated to viral clearance in patients of African and European ancestry is located upstream of *IFNL3* and is meanwhile recognized to be located within intron 1 of the *IFNL4* gene (*IFNL4* rs12979860, also known as *IL28B* rs12979860). Genetic associations were proved to be true for spontaneous clearance of HCV infection, for response to an IFN-α based therapy in patients with chronic hepatitis C and for regimens with a novel group of HCV-specific inhibitors, the direct acting antivirals [10–12].

Many of the polymorphisms in the *IFNL* gene cluster are in close linkage disequilibrium (LD). Depending on the geographical origin of the cohort it is thus challenging or even impossible to differentiate genotype associations for variants that are strongly linked. Close LD also complicates assigning the functional variant which underlies those associations. Nonetheless, for various reasons and based on special approaches, two of the polymorphisms are supposed to be the causal variants by affecting *IFNL* gene expression:

*IFNL4* rs368234815 (originally designated ss469415590) is located upstream of *IFNL3* within exon 1 of the *IFNL4* gene. Its alleles TT and ΔG determine the host’s capability to encode for IFN-λ_4_ by loss or gain of function, respectively. This locus thus harbors an intrinsic functionality by governing *IFNL4* gene expression. Moreover, by taking advantage of few rare discordant (unlinked) samples in the *IFNL4* rs12979860 and the *IFNL4* rs368234815 loci, this polymorphism was ascribed to associate with *IFNL3* transcription in polyIC stimulated peripheral blood mononuclear cells, presumably by creating a methylation motif in a CpG island [13].

*IFNL3* rs4803217 locates within the 3’untranslated region (3’UTR) of the *IFNL3* gene. Due to experiments on human hepatoma cells which were transfected with allelic *IFNL3* constructs, its T allele was shown to promote decay of *IFNL3* mRNA by two mechanisms [14]. First, the T allele was demonstrated to favor AU-rich element (ARE)-mediated decay (AMD), a post-transcriptional control which applies preferentially to genes important in immunity including many IFNs [15]. Second, the *IFNL3* rs1803217 T allele enabled repression of *IFNL3* expression by two so-called ‘myomiR’ microRNAs, miR-208b and miR-499, which are encoded within introns of myosin heavy chain (*MYH*) genes 7 and 7B, respectively, and which are co-expressed with their corresponding myosin genes. *MYH* and myomiR transcripts were shown to be inducible by HCV in human hepatoma cells. They were also found to be expressed to higher levels in some liver biopsy specimens from chronic hepatitis C patients compared to non-infected donor liver tissue [14]. As the expression of myosin genes and myomiRs is restricted to cardiac and slow skeletal muscle, it is supposed that they might be expressed ectopically in the liver in HCV infection and may affect hepatic *IFNL3* mRNA stability.

This investigation aimed at validating the impact of *IFNL3* rs4803217 and *IFNL4* rs368234815 genotypes on hepatic *IFNL3* mRNA expression in clinical samples in chronic hepatitis C in man. Furthermore, it intends to elaborate the role of hepatic *MYH7* and *MYH7B* transcript and corresponding myomiR expression on it.

## Patients and Methods

### Patients

Liver biopsy specimens were obtained from a total of 113 patients of Western European descent with chronic hepatitis C (n = 57; see Table 1), chronic hepatitis B virus (HBV) infection (n = 18; 14 males, four females; mean age 33.2 ± 11.0 years), non-viral liver diseases (n = 26; 15 males, 11 females; mean age 47.5 ± 13.4 years) and patients in whom liver disease could be ruled out by biopsy (n = 12; six males and females each; mean age 48.1 ± 18.3 years). Thus, the latter group can be referred to as ‘healthy liver tissues’. Tissues samples were snap frozen in liquid nitrogen and stored at -80°C until further use.

**Table 1:** Hepatitis C patient characteristics.

	All	*IFNL3* rs4803217 genotype			p-value
		GG	GT	TT	
Number of patients [n]	57	22	25	10	
Gender female/male [n]	24/33	5/17	16/9	3/7	0.0116
Age [mean ± SD]	46.5 ± 12.8	42.7 ± 14.5	49.3 ± 11.6	46.9 ± 11.5	0.1687
HCV types 1a/1b/1a+1b/2/3 [n]^a^	10/37/2/2/5	6/11/0/0/4	3/19/1/1/1	1/7/1/1/0	0.2023
ALT [U/l]	81 ± 69	114 ± 83	68 ± 60	55 ± 31	0.0472
AST [U/l]	57 ± 56	79 ± 73	43 ± 37	46 ± 46	0.0824
γ-GT [U/l]	74 ± 105	63 ± 64	79 ± 137	81 ± 81	0.2575
Inflammatory activity [n]^b^ mild/moderate/severe	29/26/1	7/13/1	17/8/0	5/5/0	0.1614
Fibrosis [n]^b^ absent/mild/moderate/severe/cirrhosis	12/28/9/6/1	5/10/3/2/1	5/15/2/3/0	2/3/4/1/0	0.4564
Steatosis [n]^b^ absent/mild/moderate/severe	30/17/6/3	13/8/0/0	12/7/4/2	5/2/2/1	0.3384

^a^number of patients with known viral genotype is 56

^b^number of patients with known histology is 56

Diagnosis of chronic HCV infection was based on detection of HCV-specific antibodies and HCV RNA in serum for more than six months. Chronicity of liver disease was also confirmed by histological examination. Patients with other viral infections and patients with continued alcohol or drug abuse were excluded from this group. Diagnosis of chronic HBV infection was based on detection of HBs antigen, anti-HBc antibodies and HBV-DNA. Histopathological findings and diagnoses of patients with non-viral liver diseases comprised primary biliary cirrhosis (n = 2), autoimmune hepatitis (n = 3), hemochromatosis (n = 3), steatosis or steatohepatitis (n = 2), non-specific fibrosis (n = 1), ethanol-induced liver disease or cirrhosis (n = 2), non-Hodgkin lymphoma (n = 3), hepatopathy (n = 3), liver metastases (n = 2), hyperlipoproteinemia (n = 1), cholestasis (n = 2) or non-specific hepatitis (n = 1). Healthy liver tissue comprised samples taken from patients with slightly elevated serum transaminase activities (n = 4) or increased isolated γ-GT activities (n = 2), exclusion of focal nodular hyperplasia (n = 1) as well as baseline biopsies from donor livers assessed during the course of living liver donation (n = 5).

Two myocardial control samples were acquired from left ventricular tissue of explanted hearts directly in the operating room during surgical procedures and immediately placed in precooled cardioplegic solution (in mmol/L: NaCl 110, KCl 16, MgCl_2_ 16, NaHCO_3_ 16, CaCl_2_ 1.2, glucose 11).

### Ethics Statement

This study was approved by the ethics committee of the University of Goettingen, Goettingen, Germany, on 13 July 2010 and conformed to the current ethical principles of the Declaration of Helsinki. Informed written consent was obtained from each patient.

Myocardial control samples were obtained during heart transplantation from explanted hearts of two patients with end-stage heart failure due to dilated cardiomyopathy (DCM) in compliance with the declaration of Helsinki. This study was approved by the ethics committee of the University of Goettingen, Goettingen, Germany, on 31 Sep 2000. Informed consent was obtained in written.

### Isolation of nucleic acids

Genomic DNA and total cellular RNA were simultaneously isolated from liver biopsy specimens, which had been disrupted and homogenized by a rotor-stator homogenizer, by using the AllPrep DNA/RNA Mini Kit (Qiagen) and following a protocol provided by the manufacturer for the purification of total RNA including small RNAs. According to this protocol, precipitation was carried out by taking up the lysate in 1.5 volumes of absolute ethanol instead of adding one volume of 70% ethanol as it is a standard for the purification of total RNA >200 nucleotides. RNA concentration and purity were checked in a photometer at λ = 260nm and λ = 280nm. RNA integrity was ascertained by 0.6% agarose gel electrophoresis.

### Determination of HCV genotype

HCV genotyping was performed by using the Innolipa HCV II line probe assay (Innogenetics).

### Genotyping

*IFNL3* rs4803217 genotyping was performed according to an assay described by McFarland *et al*. [14]. In brief, a region spanning the *IFNL3* locus was pre-amplified from genomic DNA using the primers given. A 1:1,000 dilution of the amplicon of 3,308 nt in length was then subjected to genotyping using a 5’nuclease assay as described [14]. *IFNL4* rs368234815/ss469415590 genotyping was carried out as described by Prokunina-Olsson *et al*. [8]. All reactions (10 μl) and analyses were carried out in the sequence detection system StepOne Plus (Applied Biosystems, Darmstadt, Germany) using the TaqMan Genotyping Master Mix (life technologies) according to the supplier’s instructions.

### Quantification of hepatic gene expression

Total cellular RNA was reverse transcribed by using the QuantiTect Reverse Transcription Kit (Qiagen) with hexamer primers only (120 pmol). Complementary DNA (cDNA) corresponding to 6.4 ng of RNA was amplified using commercially available validated assays for *GAPDH* (Hs99999905_m1), *MYH7* (Hs01110632_m1) and *MYH7B* (Hs00293096_m1) and TaqMan Universal Master Mix (life technologies) in 10 μl reactions.

### Quantification of hepatic miRNA expression

Hepatic miRNA was quantified by using commercially available TaqMan MicroRNA assays according to the instructions of the supplier (life technologies). In brief, cDNA reactions were carried out by applying TaqMan Micro RNA Reverse Transcription Kit on 5 ng of total RNA. 0.7 μl of the cDNA was transferred to singleplex TaqMan MicroRNA assays mmu-mir-499 and (assay ID 001352) and hsa-miR-208B (assay ID 002290) for quantifying mature mir499 and mir208B, respectively, and to the TaqMan MicroRNA control assay RNU6B (assay ID 001093) data were normalized for. All reactions (10 μl) and analyses were carried out in the sequence detection system StepOne Plus (Applied Biosystems, Darmstadt, Germany).

### Statistical analysis

Univariate comparisons were calculated with the PC-STATISTIK software package version 4.0 (Hoffmann-Software Giessen, Germany). *P*-values of less than 0.05 were considered statistically significant.

Exact test for deviation from Hardy-Weinberg equilibrium was performed using the online calculator provided by the Institute of Human Genetics, Helmholtz Center Munich, Germany (http://www.ihg.gsf.de/cgi-bin/hw/hwa1.pl). Cubic exact solution web software CubeX (http://www.oege.org/software/cubex/) was used to determine LD coefficients D’ and r^2^ [16]. LD map analysis was performed by applying MIDAS v1 software (http://www.genes.org.uk/software/midas).

## Results

### Characterization of the hepatitis C cohort

A total of 57 chronic hepatitis C patients of Western Europe descendent were included into this investigation. *IFNL3* rs4803217 and *IFNL4* rs368234815 genotyping revealed genotype distributions of 22:25:10 (GG:GT:TT) and 20:29:8 (TT/TT:TT/ΔG:ΔG/ΔG) with minor allele frequencies (MAF) of 0.39 both. Both distributions meet Hardy-Weinberg equilibrium (p = 0.5352 and p = 0.6250, respectively). The favorable major allele G of *IFNL3* rs4803217 was closely correlated to the favorable *IFNL4* disrupting allele TT of *IFNL4* ss469415590. 5/57 patients were found to be discordant at these two loci, yielding LD coefficients of D’ = 0.887 and *r* ^*2*^ = 0.7862. By including all available data on the total cohort (hepatitis C patients and controls) LD mapping revealed D’ coefficients of D’ = 0.90 for rs4803217 and rs368234815, D’ = 0.92 for rs4803217 and rs12979860 and D’ = 0.96 for rs368234815 and rs12979860, respectively (data not shown). Hepatitis C patient clinical characteristics including HCV genotypes, serum transaminase activities and histology are summarized in Table 1. An analysis of the patients’ clinical data with regard to *IFNL3* rs4803217 GG, GT, and TT genotypes revealed no significant differences except for a higher proportion of females among heterozygotes and slightly higher serum ALT and AST activities in *IFNL3* rs4803217 G homozygotes (Table 1). The latter relation becomes more obvious when G homozygotes were compared to T allele carriers (p = 0.0068 and p = 0.0131, Wilcoxon rank test, for ALT and AST activities, respectively) and is consistent with the well-known association of other favorable *IFNL3* genotypes and high ALT activities.

### Hepatic *IFNL3* mRNA expression in chronic hepatitis C with regard to *IFNL3* rs4803217 and *IFNL4* rs368234815 genotypes

**Fig 1:**
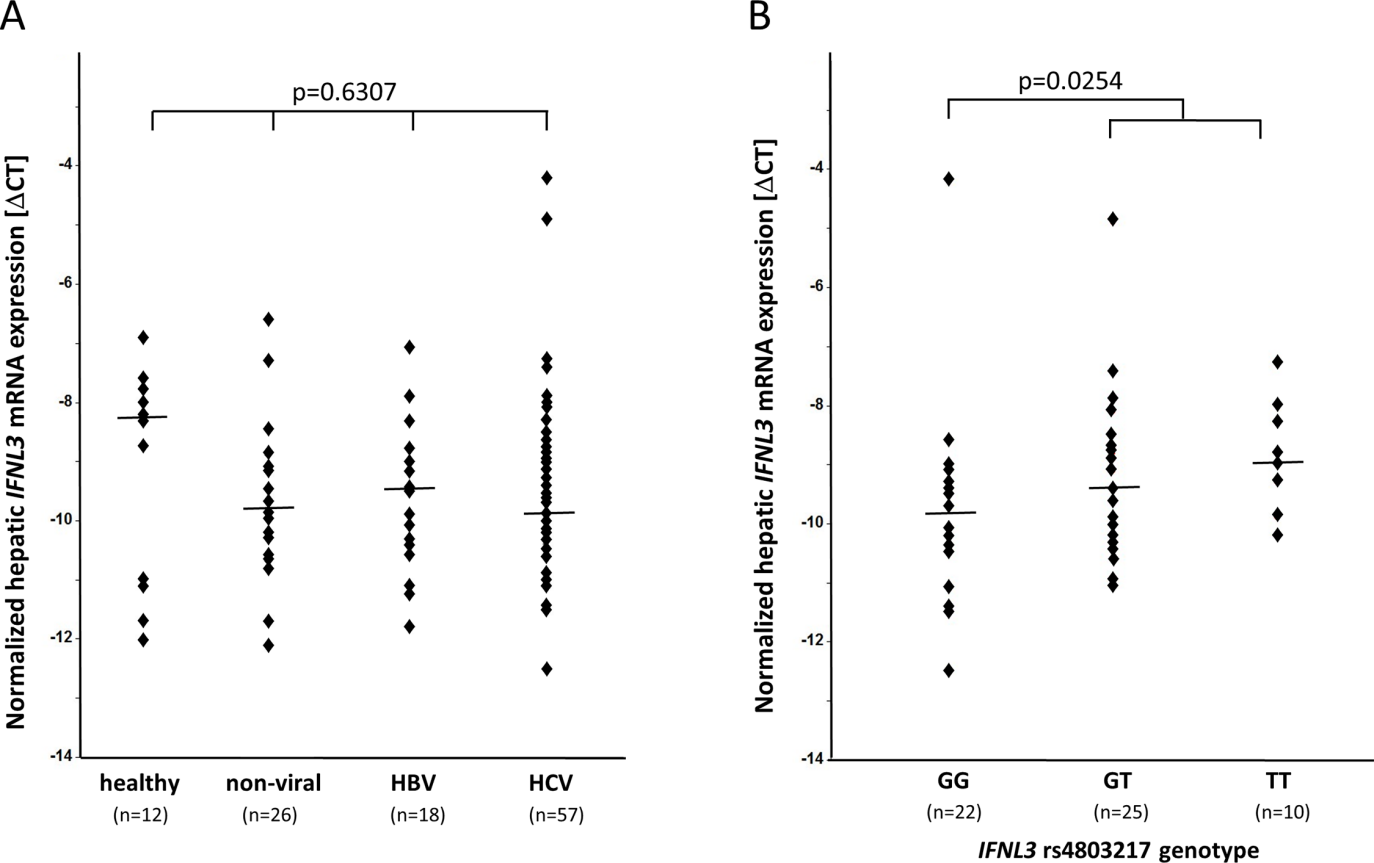
*IFNL3* transcript expression in human liver samples. (A) Liver tissue samples taken from patients without any liver condition (healthy controls), with liver diseases of non-viral etiology, or with chronic HBV or HCV infection were quantified for *IFNL3* gene expression by a 5’-nuclease assay as outlined in the Patients and Material section. Data were normalized to *GAPDH* transcripts as a reference. Kruskal-Wallis analysis revealed a lack of a significant difference between the four groups. (B) Data from hepatitis C patients were analyzed with regard to their *IFNL3* rs4803217 genotypes. *IFNL3* rs4803217 T allele carriers presented with higher *IFNL3* transcript numbers than GG homozygotes (Wilcoxon rank test). Medians and p-values are indicated.

*IFNL2* and *IFNL3* constitute highly homologous paralogues with nearly identical coding, non-coding and flanking sequences. A previous analysis of hepatic *IFNL2/IFNL3* transcripts in chronic hepatitis C patients was carried out covering them both and did not reveal any induction by chronic HCV infection in patient livers [17]. To discriminate between *IFNL2* and *IFNL3* and to specifically quantify hepatic *IFNL3* transcripts, we now applied an assay which was described by Bibert *et al*. [13]. Likewise, we found *IFNL3* transcription not to be activated in tissue specimens from chronic hepatitis C patients (n = 57) when comparing it to tissue samples from individuals with healthy livers (n = 12), to those with liver diseases of non-viral etiology (n = 26) or to those with chronic hepatitis B (n = 18) (Fig 1A).

Among patients with chronic hepatitis C, *IFNL3* rs4803217 T allele carriers—supposed to be prone to *IFNL3* transcript decay—were not found to be attenuated in *IFNL3* transcript expression when compared to G homozygotes (Fig 1B). Of note, an even rather opposed tendency was observed (p = 0.0254, Wilcoxon rank test) (Fig 1B). A sensitivity analysis deleting 7 patients with HCV non-1 genotypes affirmed this tendency (p = 0.0462, data not shown). The same pattern delineates for the *IFNL4* rs368234815 ΔG allele having described to create a methylation site putatively impairing transcription (p = 0.0487, TT/TT vs ΔG allele carriers, data not shown).

### Myosin and myomiR transcript expression in human liver samples

**Fig 2:**
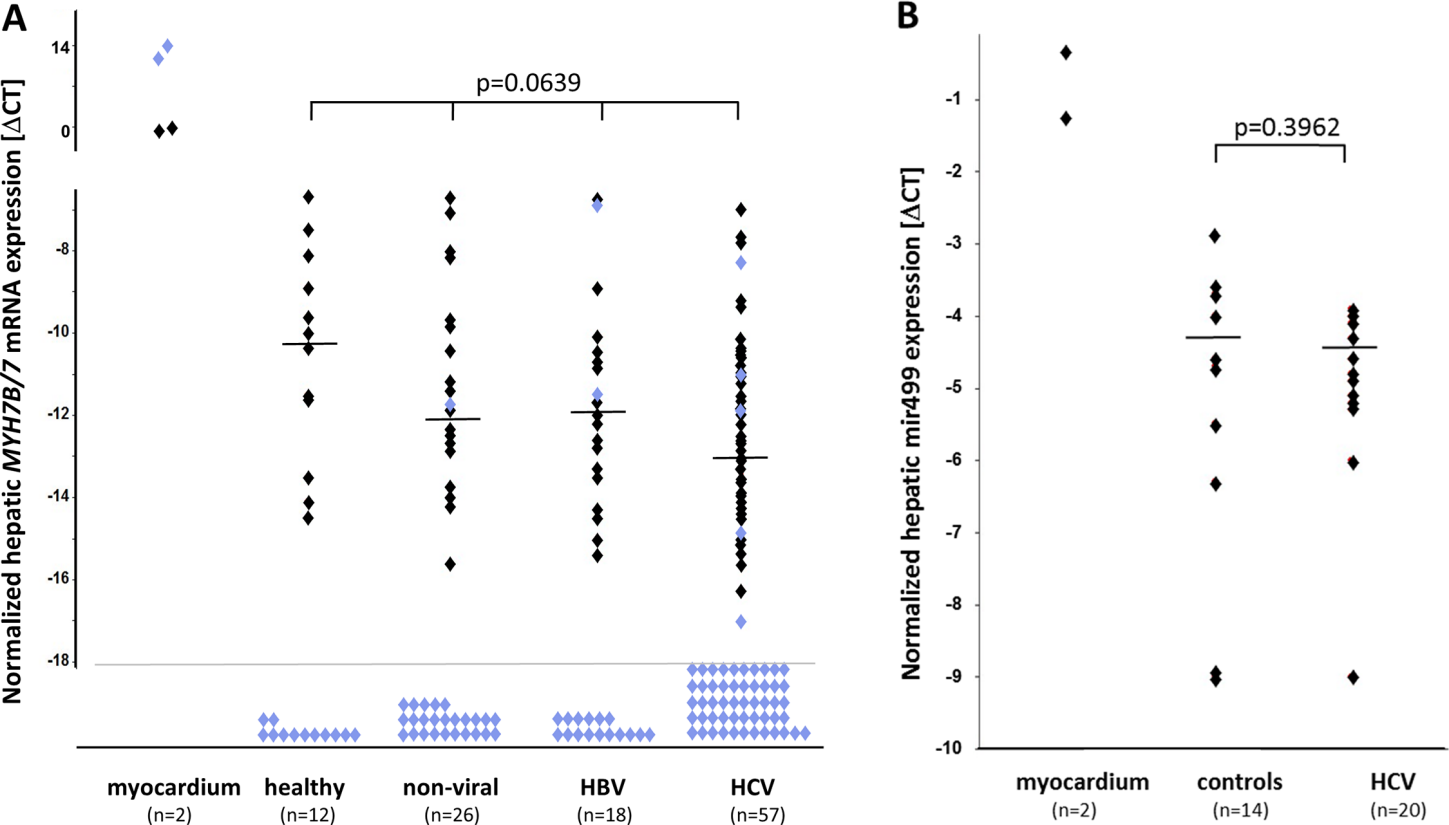
Expression of myosin and myomiR transcripts in human liver specimens. (A) Hepatic RNA preparations were quantified for *MYH7B* (black symbols) and *MYH7* (blue symbols) transcript expression by commercially available assays. Two samples from human myocardium served as a positive control. Data are normalized to *GAPDH* mRNA as a reference. Groups of patients were not found to differ significantly with regard to *MYH7B* expression (Kruskal-Wallis analysis). (B) Those samples which had been prepared for simultaneous extraction of microRNAs were quantified for mir499 by a commercially available assay as outlined in the Material and Method section. Two samples of myocardium served as a positive control. Data were normalized to RNU6B as a reference. Wilcoxon rank test revealed absence of a significant difference in mir499 expression in hepatitis C patients and controls. Medians and p-values are indicated.

To examine whether HCV infection provokes ectopic induction of myosin genes in the liver, liver biopsy specimens from patients with chronic hepatitis C were compared to those from patients with liver diseases unrelated to viral infection and to those from liver-healthy individuals with regard to *MYH7* and *MYH7B* transcript expression. Tissue from human myocardium (two patients) served as a positive control. Constitutive levels of *MYH7B* transcripts were detectable in all patient samples with no significant difference in its expression among the four patient groups (Fig 2A). Hepatitis C patients were not found to express significantly higher amounts than the respective controls either (Fig 2A). Again, a sensitivity analysis deleting hepatitis C patients with non-1 viral genotypes confirmed the lack of a relationship between hepatic *MYH7B* expression and chronic HCV infection (p = 0.1240, data not shown). To the contrary, *MYH7* mRNA was found to be below the limit of detection or in a grey range with both detectable and non-detectable amplification signals in multiplicate reactions in most of the specimens (94/113 and 10/113, respectively) (Fig 2A). Thus, by means of transcript quantification we did not find any evidence for an activation of *MYH7B* expression above a constitutive level nor of *MYH7* being mostly below the limit of detection in chronic HCV infection.

Data on myosin mRNA expression were corroborated by data on myomiR expression. While myomiR mir499 which is encoded within the *MYH7B* gene was easily detectable in all of those samples that had been extracted according a protocol to inclose microRNAs (Fig 2B), mir208B which is encoded within the *MYH7* gene was not detected (data not shown). As with *MYH7B* gene expression, mir499 expression was not found to differ significantly between patient groups (Fig 2B).

Apart from our finding of non-inducibility of myosin genes by HCV infection *in vivo*, also the lack of *MYH7* expression in liver samples appeared to contrast findings by McFarland *et al*. We were wondering whether the procedure of obtaining liver tissue might have affected results. While McFarland *et al*. compared HCV-infected liver tissue obtained by percutaneous liver punctation to non-HCV infected tissue from donor livers, liver tissues from our investigation was obtained either by percutaneous punctation (n = 21) or by a direct punctation of the liver during laparascopic inspection (n = 83) irrespective of disease etiology according to patient records. We hypothesized that percutaneous liver punctation might originate in a sample containing traces of muscle tissue. Our analysis revealed that most of the tissues that were obtained during laparoscopy did not contain detectable amounts of *MYH7* mRNA (76/83) while samples with detectable amounts of *MYH7* transcripts or only marginal *MYH7* expression were mostly obtained by percutaneous punctation (10/17) (Table 2).

**Table 2:** Detection of *MYH7* mRNA with regard to the procedure of biopsy sampling.

*MYH7* mRNA	Biopsy sampling	
	percutaneously	during laparoscopy
detected (n)	6	2
grey range^a^ (n)	4	5
non-detected (n)	11	76

p<0.0001, χ^2^-test

^a^Grey range defines results with both detectable and non-detectable amplification signals in multiplicate reactions

## Discussion

By applying a gene expression assay being specific for *IFNL3* and discriminating the highly homologous paralogue *IFNL2*, we demonstrate a lack of an activation of hepatic *IFNL3* gene expression in clinical liver biopsy samples from patients with chronic hepatitis C when compared to liver-healthy individuals, patients with liver diseases of non-viral etiology, or patients with chronic hepatitis B. This result complements own previous findings on the lack of hepatic *IFNL2/3* gene activation in chronic HCV infection in comparison to non-viral liver diseases in man [17].

Given an only constitutive level of expression of *IFNL3* in the liver of hepatitis C patients, its level was not found to be higher in *IFNL4* rs368234815 TT homozygotes than in ΔG allele carriers. Bibert *et al*. [13] had proposed a functional role of *IFNL4* rs368234815 in *IFNL3* gene expression as by stimulating peripheral blood mononuclear cells with polyIC they found significantly higher expression of *IFNL3* in TT homozygotes than in ΔG homozygotes. They provided evidence for the introduction of a methylation site into a CpG motif by the *IFNL4* rs368234815 ΔG variant. This proposed epigenetic mechanism might not apply to the same extent to human liver tissue. Interestingly, we observed even an opposing relationship as ΔG allele carriers appeared to reach higher numbers of hepatic *IFNL3* transcripts than TT homozygotes. In view of a close LD between *IFNL4* rs368234815 and *IFNL4* rs12979860, this finding is in line with a recent finding by Noureddin *et al*. [18]. The authors demonstrated slightly higher *IFNL2/IFNL3* mRNA expression in the liver of hepatitis C patients carrying the minor non-favorable *IFNL4* rs12979860 C alleles [18].

The constitutive level of hepatic *IFNL3* expression was not found to be attenuated in *IFNL3* rs4803217 CT/TT genotypes either, reflecting the close LD between the two loci in our cohort. A functionality of this SNP was advanced by McFarland *et al*. who demonstrated stronger AMD and stronger microRNA-mediated decay in human hepatoma cells transfected with minor allele *IFNL3* 3’UTR as compared to *IFNL3* 3’UTR major allele reporter constructs. The microRNA-mediated decay was suggested to be driven by an induction of *MYH7B* and *MYH7* and their corresponding myomiRs mir499 and mir208B by HCV. This assumption was based on the induction of *MYH* genes in HCV-infected hepatoma Huh-7 cells. The authors also demonstrated myosin gene expression analyses of some liver tissue specimens. Different to the finding by McFarland *et al*. our data based on a total of 113 liver biopsy specimens revealed a lack of *MYH7B/7* gene activation in chronic hepatitis C with even *MYH7* being below the limit of detection. As the regulatory region of *MYH7B* but not that of *MYH7* contains binding sites for STAT1 and STAT3 (http://genome.ucsc.edu, genome assembly Feb. 2009 (GRCh37/hg19)) [19], constitutive levels of IFNs might account for differential hepatic expression of *MYH7B* and *MYH7*. The apparent discrepancy in findings on the activation of myosin and myomiR expression in chronic hepatitis C might be due to the different sources of liver tissue. While McFarland *et al*. compared unused donor liver to liver biopsy specimens from hepatitis C patients we used biopsy specimens only, from both hepatitis C patients and controls. Alternatively, percutaneous sampling could have biased results as percutaneous sampling might entrain traces of muscle tissue. To test this assumption, we compared biopsy samples obtained by direct punctation of the liver in the course of laparoscopic inspection to tissue samples that were obtained by percutaneous liver needle biopsy. As we found *MYH7* expression preferentially in those samples that had been obtained by percutaneous needle biopsy, data suggest that traces of muscle contaminate these samples and argue against a hepatic induction due to HCV infection. This finding is in accordance with the finding that hepatic *MYH7B* mRNA expression does not differ significantly between hepatitis C patients and the respective controls, meaning that neither hepatic *MYH7B* nor *MYH7* gene expression appears to be activated in chronic HCV infection.

Different to *IFNL1-3* transcripts, we recently demonstrated *IFNL4* transcripts to be clearly activated in chronic HCV infection in patient livers compared to non-HCV infected controls [20,21]. Generation of the *IFNL4* protein is enabled by the *IFNL4* rs368234815 ΔG variant, while the *IFNL4* rs368234815 TT allele prevents translation by causing a frameshift [8]. This intrinsic, natural functionality is likewise assumed to be the causal variant underlying the association between *IFNL* genotypes and HCV clearance [8,22]. By taking advantage of a low LD between *IFNL4* rs368234815 or *IFNL3* rs4803217 in African American individuals, a recent study identified *IFNL4* rs368234815 to be the primary polymorphism determining viral clearance [23]. This intrinsic functionality might combine with further roles of this polymorphism, e.g. in epigenetic regulation of *IFNL3* in peripheral blood mononuclear cells. Our study, yet, could not reveal any evidence for the proposed role of *IFNL4* rs368234815 in hepatic *IFNL3* gene expression, neither for *IFNL3* 3’UTR rs4803217.

In conclusion, the lack of an induction of *IFNL3* by HCV in the liver of patients with chronic hepatitis C, the lack of an attenuation of basal *IFNL3* transcript expression in *IFNL3* rs4803217 or *IFNL4* rs368234815 minor allele carriers, and the lack of an induction of myosin genes and their corresponding myomiRs argue against *IFNL3* rs4803217 or *IFNL4* rs368234815 variations to be causally related to clearance of HCV infection by modulating hepatic *IFNL3* expression. Our findings, moreover, point at the importance of complementing functional mechanistic evidence with authentic clinical data to unravel the essential mechanisms for persistence and resolution of HCV infection operating *in vivo* in man.
